# Internet-delivered cognitive behavioral therapy for tinnitus compared to Internet-delivered mindfulness for tinnitus: a study protocol of a randomized controlled trial

**DOI:** 10.1186/s13063-023-07299-9

**Published:** 2023-04-12

**Authors:** Jonas Eimontas, Goda Gegieckaitė, Irena Asačiova, Nikol Stičinskaitė, Livija Arcimavičiūtė, Dovilė Savickaitė, Donata Vaitiekūnaitė-Zubriakovienė, Marius Polianskis, Jennifer Gans, Eldre Beukes, Vinaya Manchaiah, Gerhard Andersson, Eugenijus Lesinskas

**Affiliations:** 1 Department of Clinical Psychology, Institute of Psychology, Vilnius University, Vilnius, Lithuania; 2 Institute of Psychology, Vilnius University, Vilnius, Lithuania; 3 Faculty of Medicine, Vilnius University, Vilnius, Lithuania; 4 Vilnius University Hospital Santaros Klinikos, Vilnius, Lithuania; 5 Clinic of Ear, Nose, Throat and Eye Diseases, Institute of Clinical Medicine, Faculty of Medicine, Vilnius University, Vilnius, Lithuania; 6 Mindfulness Based Tinnitus Stress Reduction (MBTSR), San Francisco, CA USA; 7 Vision and Hearing Sciences Research Centre, Anglia Ruskin University, Cambridge, UK; 8 Virtual Hearing Lab, Collaborative Initiative Between University of Colorado School of Medicine and University of Pretoria, Aurora, CO USA; 9 Department of Otolaryngology-Head and Neck Surgery, University of Colorado School of Medicine, Aurora, CO USA; 10 UC Health Hearing and Balance, University of Colorado Hospital, Aurora, CO USA; 11 University of Pretoria, Pretoria, South Africa; 12 Department of Speech-Language Pathology and Audiology, University of Pretoria, Gauteng, South Africa; 13 Department of Speech and Hearing, Manipal College of Health Professions, Manipal Academy of Higher Education, Manipal, India; 14 Department of Behavioural Sciences and Learning, Department of Biomedical and Clinical Sciences, Linköping University, Linköping, Sweden; 15 Department of Clinical Neuroscience, Karolinska Institute, Stockholm, Sweden

**Keywords:** Internet-delivered interventions, Tinnitus, Cognitive behavioral therapy, CBT, ICBT, Mindfulness, MBTSR, iMBTSR, RCT, Tinnitus distress

## Abstract

**Background:**

Tinnitus affects around 15% of the population and can be a debilitating condition for a sizeable part of them. However, effective evidence-based treatments are scarce. One recommended treatment for tinnitus is cognitive behavioral therapy which has been found to be effective when delivered online. However, more treatments including mindfulness-based interventions have been studied recently in an attempt to facilitate the availability of effective treatments. There are promising findings showing great effects in reducing tinnitus-induced distress and some evidence about the efficacy of such intervention delivered online. However, there is a lack of evidence on how these two treatments compare against one another. Therefore, the aim of this study will be to compare Internet-delivered cognitive behavioral therapy for tinnitus against an Internet-delivered mindfulness-based tinnitus stress reduction intervention in a three-armed randomized controlled trial with a waiting list control condition.

**Methods:**

This study will be a randomized controlled trial seeking to recruit Lithuanian-speaking individuals suffering from chronic tinnitus. The self-report measure Tinnitus Handicap Inventory will be used. Self-referred participants will be randomized into one of three study arms: Internet-delivered cognitive behavioral therapy, Internet-delivered mindfulness-based tinnitus stress reduction intervention, or a waiting-list control group. Post-treatment measures will be taken at the end of the 8-week-long intervention (or waiting). Long-term efficacy will be measured 3 and 12 months post-treatment.

**Discussion:**

Internet-delivered interventions offer a range of benefits for delivering evidence-based treatments. This is the first randomized controlled trial to directly compare Internet-delivered CBT and MBTSR for tinnitus in a non-inferiority trial.

**Trial registration:**

ClinicalTrials.gov NCT05705323. Registered on January 30, 2023.

## Administrative information

Note: The numbers in curly brackets in this protocol refer to the SPIRIT checklist item numbers. The order of the items has been modified to group similar items (see http://www.equator-network.org/reporting-guidelines/spirit-2013-statement-defining-standard-protocol-items-for-clinical-trials/).

**Table:** 

Title {1}	Internet-delivered cognitive behavioral therapy for tinnitus compared to Internet-delivered mindfulness for tinnitus: a study protocol of a randomized controlled trial
Trial registration {2a and 2b}.	This study has been registered in clinicaltrials.gov. ClinicalTrials.gov Identifier: NCT05705323 https://beta.clinicaltrials.gov/study/NCT05705323
Protocol version {3}	Protocol version 1.0, 2022–11-13
Funding {4}	This project has received funding from the Research Council of Lithuania (LMTLT), agreement No S-PD-22–178.
Author details {5a}	Jonas Eimontas, PhD, Department of Clinical Psychology, Institute of Psychology, Vilnius University, Vilnius, Lithuania, jonas.eimontas@fsf.vu.lt (Corresponding author) Goda Gegieckaitė, PhD, Department of Clinical Psychology, Institute of Psychology, Vilnius University, Vilnius, Lithuania, goda.gegieckaite@fsf.vu.lt Irena Asačiova, Institute of Psychology, Vilnius University, Lithuania, irena.asaciova@fsf.stud.vu.lt Nikol Stičinskaitė, Institute of Psychology, Vilnius University, Lithuania, nikol.sticinskaite@fsf.stud.vu.lt Livija Arcimavičiūtė, Institute of Psychology, Vilnius University, Lithuania, livija.arcimaviciute@fsf.stud.vu.lt Dovilė Savickaitė, Institute of Psychology, Vilnius University, Lithuania, dovile.savickaite@fsf.stud.vu.lt Donata Vaitiekūnaitė-Zubriakovienė, 1. Faculty of Medicine, Vilnius University. 2. Vilnius University Hospital Santaros klinikos, Vilnius Lithuania. donata.vaitkunaite@santa.lt Marius Polianskis, Clinic of Ear, Nose, Throat and Eye Diseases, Institute of Clinical Medicine, Faculty of Medicine, Vilnius University, Vilnius, Lithuania, marius.polianskis@santa.lt Jennifer Gans, PhD, Mindfulness Based Tinnitus Stress Reduction (MBTSR), San Francisco, CA, jg@mindfultinnitusrelief.com Eldre Beukes, PhD, 1. Vision and Hearing Sciences Research Centre, Anglia Ruskin University, Cambridge, UK. 2. Virtual Hearing Lab, Collaborative Initiative between University of Colorado School of Medicine and University of Pretoria, Aurora, CO, USA, eldre.beukes@aru.ac.uk Vinaya Manchaiah, AuD, MBA, PhD, 1. Department of Otolaryngology-Head and Neck Surgery, University of Colorado School of Medicine, Aurora, CO, USA. 2. UC Health Hearing and Balance, University of Colorado Hospital, Aurora, CO, USA. 3. Virtual Hearing Lab, Collaborative Initiative between University of Colorado School of Medicine and University of Pretoria, Aurora, CO, USA, and University of Pretoria, Pretoria, South Africa 4. Department of Speech-Language Pathology and Audiology, University of Pretoria, Gauteng, South Africa. 5. Department of Speech and Hearing, Manipal College of Health Professions, Manipal Academy of Higher Education, Manipal, India, vinaya.manchaiah@cuanschutz.edu Gerhard Andersson, PhD in Clinical Psychology and in Otorhinolaryngology, Department of Behavioural Sciences and Learning, Department of Biomedical and Clinical Sciences, Linköping University, Linköping, Sweden; Department of Clinical Neuroscience, Karolinska Institute, Stockholm, Sweden, gerhard.andersson@liu.se Eugenijus Lesinskas, MD PhD, Clinic of Ear, Nose, Throat, and Eye Diseases, Institute of Clinical Medicine, Faculty of Medicine, Vilnius University, Vilnius, Lithuania, eugenijus.lesinskas@mf.vu.lt
Name and contact information for the trial sponsor {5b}	Vilnius University, email address: infor@cr.vu.lt
Role of sponsor {5c}	Sponsor and funder have no role in the study design, collection, management, analysis, and interpretation of the data and writing of the report. Principle investigators have full control of decision to submit the report for publication.

## Introduction

### Background and rationale {6a}

Tinnitus is the perception of an auditory sensation, perceivable without the presence of an external sound. The prevalence of tinnitus varies widely around the world, but it is estimated to affect approximately 10–15% of the population [1]. A recent pan-European study found that the prevalence of tinnitus was 14.7% and bothersome tinnitus 6% [2]. The same study also found that the occurrence of tinnitus becomes more prevalent as age increases and hearing deteriorates. There is still limited knowledge of the causal risk factors of tinnitus; however, there is some evidence for hearing loss and other physical conditions as well as depression being risk factors [3]. Additionally, the severity of tinnitus symptoms directly affects the utilization of healthcare resources for tinnitus management. The severity of tinnitus can be influenced by factors such as tinnitus awareness throughout the day, hearing loss, self-reported depression or anxiety, perceived loudness, education level, presence of additional physical symptoms, and variability in pitch and loudness [4, 5]. Some specific maladaptive psychological coping like avoidance and non-acceptance and negative cognitions about tinnitus has been suggested to be possible mechanisms of increased suffering from bothersome tinnitus [6–9]. Untreated bothersome tinnitus can significantly reduce the quality of life and may affect many other daily life aspects like sleep, concentration, and mental health [10]. Fortunately, psychological interventions can be effective in dealing with tinnitus-related disturbances.

### Cognitive behavioral therapy for tinnitus

Studies have found that cognitive behavioral therapy (CBT) is the most effective psychosocial treatment for tinnitus [11, 12]. Despite its established efficacy, CBT is very rarely offered to those experiencing distressing tinnitus [13]. CBT for tinnitus focuses on mechanisms thought to be behind the increased stressful reaction to tinnitus and disruption of habituation of tinnitus—negative interpretation of tinnitus, increased sympathetic arousal, selective attention and monitoring of tinnitus, and avoidance of tinnitus [13, 14]. Therefore, CBT for tinnitus usually includes elements aimed to alter these processes like psychoeducation and cognitive restructuring of negative beliefs about tinnitus, applied relaxation, and behavioral change, especially exposure to tinnitus as well as emotional reactions in response to tinnitus [13, 15, 16]. CBT focuses on cognitive and behavioral responses to tinnitus and aims to minimize distress caused by tinnitus rather than the symptom of tinnitus itself.

Practitioners treating patients with bothersome tinnitus are increasingly seeking new ways to increase access to cognitive behavioral therapy (CBT) due to its efficacy and success in treating tinnitus [17]. The creation of an Internet-based CBT intervention for tinnitus (ICBT) [15] is one such strategy, including the treatment components of face-to-face CBT but in a text form online and has additional features like illustrations and videos; it usually lasts 9 weeks, introduces new modules covering different topics weekly, and is often being supported by a clinician who answers questions and provides feedback on homework assignments during the whole treatment process [15]. ICBT has been found to be as effective for tinnitus as face-to-face CBT [18]. One study compared Internet-based cognitive behavioral therapy to individualized face-to-face clinical treatment in reducing distress from tinnitus, and the results showed that both interventions were equally effective in reducing tinnitus distress and the majority of tinnitus-related difficulties [19]. ICBT has also been found to be similarly effective as group CBT for tinnitus [20]. Systematic review and meta-analysis in 2019 reported results of nine randomized clinical trials (RCTs) in Europe that revealed positive outcomes and showed that ICBT was effective for adults, with improvements in anxiety, depression, quality of life, insomnia, and tinnitus distress [21]. Therefore, ICBT seems to be an effective and accessible way to provide help to people suffering from bothersome tinnitus.

Due to the fact that cognitive behavioral therapy is an effective treatment tool for helping people with tinnitus, there are also some limitations that are often discussed by researchers in such studies. The main challenge is finding a sufficient number of volunteers experiencing bothersome tinnitus. Having potentially come a long way before being able to seek audiology and otolaryngology services, some patients may wish to continue down this path and not to participate in a research study. Therefore, researchers will need to find more efficient recruitment strategies [19].

### Efficacy of mindfulness-based practice in the treatment of tinnitus

In the last decade, interest in mindfulness-based approaches as a potential management tool for tinnitus started to grow. As research on mindfulness-based interventions’ efficacy on various physical conditions, particularly chronic pain [22, 23] showed some promising results, new research incorporating mindfulness practices for tinnitus management started to come out. Kabat-Zinn in 1982 described mindfulness as a practice in the non-judgmental observation of all physical and mental events—body sensations, thoughts, and memories [24]. When explaining the potential benefit of mindfulness in the case of chronic pain, he details how through continuous practice a person develops an ability to observe without attachment an intense feeling in the body as a bare sensation, accompanying alarming thoughts—as simple mind events and this way decreasing the body’s alarm reactivity to body sensations [24]. The same benefits of practicing mindfulness can be experienced in the case of other physical and mental conditions, including tinnitus. Gans et al. describe mindfulness practice as helping those with tinnitus to notice thoughts, emotions, and body sensations as temporary events, rather than unbearable or unending sensations, therefore alleviating some of the co-occurring distress [25]. The most common and the most systematic training of mindfulness is done through mindfulness-based stress reduction (MBSR), Kabat-Zinn developed 8-week group program teaching techniques to practice non-judgmental awareness, mindfulness meditation, and acceptance of chronic conditions incorporating meditation, yoga, and psychoeducation [26]. However, teaching and promoting mindfulness practices have been incorporated into various modes of therapies and interventions, for example, mindfulness-based cognitive behavioral therapy has been developed [27].

Rademaker et al. did a systematic review of mindfulness-based interventions on tinnitus distress and found 7 studies testing some form of mindfulness-based intervention efficacy for tinnitus-related distress [28]. Previous studies were either testing mindfulness combined with cognitive therapy [29–31], interventions incorporating mindfulness as a core or additional element [32, 33], or MBSR program [25, 34]. Rademaker et al. [28] found that regardless of the type of mindfulness-based intervention reductions in tinnitus distress after the intervention was observed. However, these studies differ in specifics of their design and their findings, for example, how effective they were in secondary outcomes and whether effects were maintained at follow-ups. Rademaker et al. [28] reported that there was no effect of MBIs observed for depression and anxiety in tinnitus patients; however, this might be due to low scores of depression and anxiety of participants at pre-treatment.

It was hypothesized that mindfulness practice could help lessen judgment and reactivity to difficult body sensations and cognitions and therefore could be helpful in decreasing tinnitus distress [24, 25, 34]. Other studies of mindfulness-based interventions for tinnitus showed a more detailed view of possible mechanisms. McKenna et al.’s study results showed that after participating in mindfulness-based cognitive behavioral therapy, there were significant changes in tinnitus catastrophizing, avoidance behaviors, and tinnitus acceptance [31]. In Phillipot et al.’s study, at 3 months follow-up, the mindfulness group reported less negative emotion, irritability, and rumination than they reported before mindfulness training but already after psychoeducation session [30]. Gans et al. found improvements in participants’ mindfulness dimensions of non-judging, increase in observing, and in reactivity to inner experience [26]. Participants reported changes in how they accepted their bodies and their tinnitus experience in the qualitative part of the study [25]. The MBTSR course is designed to reduce participant’s anxiety about having tinnitus and provide accurate up-to-date education on tinnitus, while supporting a mindfulness meditation practice to relieve stress [26]. As explained by Rademaker et al. [28], these findings are in line with some cognitive conceptualizations of factors associated with bothersome tinnitus—negative cognitions, conditioned fear reaction to tinnitus, and tinnitus avoidance. Mindfulness-based interventions in helping to accept the experience of tinnitus, increasing cognitive and metacognitive awareness, and therefore decreasing negative thoughts and their effects should lower tinnitus distress [28].

However, many of the previously described studies combined mindfulness and cognitive behavioral techniques [29–31]; therefore, it is hard to exactly pinpoint the efficacy and exact mechanisms working in mindfulness versus cognitive techniques. Studies of mindfulness programs found significant reductions in the severity of tinnitus bother [25, 34], and Roland et al.’s [34] study found differences in functional connectivity within regions associated with known attention networks pre- and post-MBSR program. However, both studies were conducted without control groups and with small samples. While in Roland et al.’s [34] study participants attended a general MBSR course, Gans et al. [25] developed mindfulness-based tinnitus stress reduction (MBTSR) designed to address participants’ unique experience with tinnitus. The biggest modifications of MBSR were psychoeducation about tinnitus and psychoeducation related to the often co-occurring disorders common in people with tinnitus and guided mindfulness practices included more emphasis on awareness of sound and tinnitus perception [25]. MBTSR was recently implemented online, mirroring the in-person MBTSR program, and significant reductions in perceived stress and tinnitus self-function were found among those who finished the program [26]. This study by Gans et al. [26] investigated if there is a meaningful change in tinnitus intrusiveness after taking the Internet-delivered iMBTSR course and whether changes were retained at a 6-month follow-up. The results showed a significant reduction in tinnitus intrusiveness with the greatest gains noted between pre-treatment to mid-treatment. Gains were maintained 6 months after treatment demonstrating the efficacy of this Internet-delivered 8-week course [26].

### Internet-based mindfulness and cognitive behavioral therapy for tinnitus

While there is enough evidence for the efficacy of cognitive behavioral therapy for tinnitus and emerging evidence of mindfulness as an effective tool for managing tinnitus, previous research on mindfulness interventions for tinnitus were studying mindfulness combined with cognitive behavioral therapy techniques or had no control group to compare the efficacy to. Finally, no previous study compared mindfulness-based intervention directly to cognitive behavioral therapy for tinnitus. Testing Internet-based versions of both interventions in comparison with the control group will be another step toward facilitating wider access and a range of effective psychological interventions for tinnitus distress.

### Objectives {7}

The purpose of this study is to assess the efficacy of Internet-delivered cognitive behavioral therapy (CBT) and Internet-delivered mindfulness-based tinnitus stress reduction intervention for adults with tinnitus, by comparing their efficacy against each other and against a waiting list control group.

### Trial design {8}

This study is three-armed parallel-group randomized controlled non-inferiority trial. Participants will be randomized to all groups equally.

## Methods: participants, interventions, and outcomes

### Study setting {9}

**Fig. 1:**
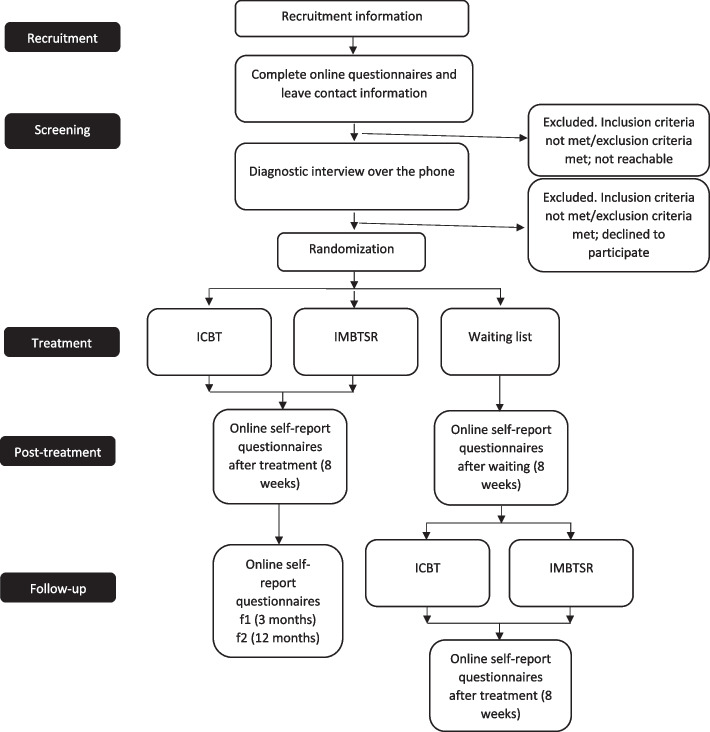
CONSORT diagram

This study will be conducted completely remotely. All screening and outcome questionnaires will be completed online and intake, and post-treatment interviews will be done on the phone. The intervention is provided via a web-based platform and is mobile device friendly. All participants who speak Lithuanian regardless of their place of residence will be considered for participation in the trial as long as they are available for an interview over the phone. The participant flowchart is shown in Fig. 1.

### Eligibility criteria {10}

All participants will have to complete self-report online questionnaires, and participants meeting all the main inclusion criteria will undergo additional intake interviews over the phone. Participants will be deemed eligible to participate in the study if they meet the following inclusion criteria: (1) 18 years and older, (2) experience tinnitus for at least 3 months, (3) scores 28 or more on Tinnitus Handicap Inventory, (4) has the ability to use a computer (or smartphone or tablet) with a connection to the internet for the duration of the study, and (5) comprehension and ability to write and speak in the Lithuanian language. Participants will be excluded from the study if they meet one of the exclusion criteria: (1) inability to allocate sufficient time for participation in an 8-week intervention, (2) significant medical or psychiatric conditions which would prevent participation, and (3) participation in other tinnitus interventions during the study.

### Who will take informed consent? {26a}

Participants will give their informed consent online by indicating a checkbox before starting to complete self-report questionnaires.

### Additional consent provisions for collection and use of participant data and biological specimens {26b}

N/A, no biological specimens will be collected.

### Interventions

#### Explanation for the choice of comparators {6b}

Internet-delivered cognitive behavioral therapy for tinnitus has accumulated a sizeable amount of evidence of its efficacy. Recent studies reported that mindfulness-based stress reduction interventions could also be effective in alleviating tinnitus-related distress. A waiting-list control will allow to compare how this particular intervention compares to those provided in previous studies.

#### Intervention description {11a}

Participants will be randomized to either of the two treatment groups or a waiting-list control group. After waiting for 8 weeks, participants in the waiting-list control group will have to choose one of the two interventions.

##### Internet-delivered cognitive behavioral therapy for tinnitus (ICBT)

The ICBT intervention for the experimental group will start after group allocation. The ICBT is based on a self-help program created by Andersson and colleagues and is designed to address the physical, emotional, and problematic effects of tinnitus for habituation [35]. The program includes key audiological principles and has been modified previously to an interactive e-learning format for better engagement [10]. The 16 recommended modules and 5 optional modules can be tailored to individual needs and are released weekly over an 8-week period. Participants are encouraged to engage with the modules and practice the suggested techniques daily. The program is comprehensive, offering a range of CBT techniques, and can be accessed online, downloaded, or printed. It includes information, videos, quizzes, diagrams, worksheets, and solutions for common problems. Participants can also use a secure messaging system to ask questions and receive feedback from their assigned program therapists.

##### Internet-delivered mindfulness-based tinnitus stress reduction.

The i-MBTSR course consists of 8 online lessons held weekly, which include videos, audio recordings, and written content covering tinnitus education, mindfulness, gentle yoga, and guided meditations [25]. Participants are required to do 30 min of daily meditation and attend a daylong practice session between the 6th and 7th week to enhance their mindfulness skills. The course aims to provide in-depth tinnitus education, reduce tinnitus-related anxiety, and teach meditation skills to help participants view tinnitus as a normal part of their sensory experience, reducing its bothersome effects. Just like the ICBT participants, individuals assigned to iMBTSR group will also have the ability to use the same secure messaging system to ask questions and receive feedback from their assigned program therapists.

#### Criteria for discontinuing or modifying allocated interventions {11b}

Participants will be instructed to notify the research staff in the event of a decline in their condition. Within 24 h, a trained clinical psychologist will conduct a thorough clinical assessment via telephonic interview. Based on the results of the evaluation, the participant may be referred for hospitalization or other necessary medical services.

#### Strategies to improve adherence to interventions {11c}

Adherence to the intervention protocols and reduction of attrition rates will be enhanced through strategies recommended by Dziura and co-authors [36]. These strategies involve data collection that does not necessitate in-person visits and frequent, guided communication throughout the trial. Participation is voluntary, and individuals have the right to withdraw without consequences. In exceptional circumstances, participants may be removed if they are unable to continue with the trial due to unforeseen reasons. The reasons for withdrawal will be documented.

#### Relevant concomitant care permitted or prohibited during the trial {11d}

Stable doses of ongoing psychopharmacological medications are permitted, but other psychological interventions are not allowed to start during the trial. If they are started after the intervention had ended, it will not result in disqualification from participating in follow-ups. After all the follow-ups, participants will be asked about receiving any other interventions during the study period, and this information will be reported with the study results.

#### Provisions for post-trial care {30}

We expect that participants will not experience any harm as a result of participating in the trial. However, participants who will show deterioration in their condition will be directed to health services accordingly.

### Outcomes {12}

We will assess the interventions’ effects on a few different areas of participants’ functioning. A primary outcome assessed will be the change in tinnitus impact on participants functioning in daily life. We will also assess as secondary outcomes changes in psychological difficulties associated with having tinnitus—depression, anxiety, and sleep difficulties. Participants’ cognitions about tinnitus and the frequency of state of mindful awareness will also be assessed as a secondary outcome in the study. Tinnitus, hearing problems, and hyperacusis will be assessed to screen the eligibility for the study and to assess any possible changes post-treatment. Finally, big five personality traits and other sociodemographic and health variables will be assessed to analyze the personal factors associated with interventions’ efficacy.

Measures included in the study and the time point for each measure will be the following.

#### Primary outcome measures

To assess the impact of tinnitus on daily life, the Tinnitus Handicap Inventory (THI) will be used [37]. Assessment with THI will take place at pre-treatment, post-treatment at week 8, and 3 and 12 months post-treatment follow-ups. Change in the score from baseline will be assessed. The THI is designed to assess the impact of tinnitus on daily life and measures tinnitus-related functional limitations and emotional and catastrophic reactions. It is also widely used in research studies to measure the change in the severity of a tinnitus handicap before and after an intervention and therefore was selected as the primary measure of the study. The THI questionnaire consists of 25 questions. Answers are scored as follows: “yes,” 4 points; “no,” 0 points; and “sometimes,” 2 points. The total score is obtained by summing the scores of all 25 questions. A higher score indicates a higher disability caused by tinnitus and a higher impact of tinnitus on a person’s daily life. A validated Lithuanian language version of THI will be used in the study [38].

Weekly monitoring of changes in tinnitus handicap severity will be done using the Tinnitus Handicap Inventory Screening version (THI-S) [39]. A screening version of THI has 10 items from the original THI and will let assess the changes throughout the duration of the program. Assessment with THI-S will be done at weeks 2, 4, and 6.

#### Secondary outcome measures

The severity of the depressive symptoms of participants will be measured with the Patient Health Questionnaire (PHQ-9) [40]. Assessment will be done pre-treatment, post-treatment (week 8), and at follow-ups (3 months and 12 months post-treatment). Change in the score from baseline will be calculated. PHQ-9 contains 9 questions answered on a 4-item Likert scale, where number 0 indicates “not at all” and 3 indicates “nearly every day.” Scores of all questions are summed for the total score, where a higher score indicates more severe symptoms.

The severity of participants’ anxiety will be assessed with Generalized Anxiety Disorder-7 (GAD-7) [41]. Assessment will take place pre-treatment, at week 8 and 3 months, and 12 months post-treatment, and change in score from baseline will be measured. The questionnaire contains 7 questions answered on a 4-item Likert scale, where number 0 indicates “not at all” and 3 indicates “nearly every day.” A higher score indicates more severe symptoms.

The insomnia symptomatology of participants will be assessed with Insomnia Severity Index (ISI) [42]. Time points for assessments will be pre-treatment, post-treatment at week 8, and 3 months and 12 months post-treatment. The questionnaire consists of 7 questions answered on a 5-item Likert scale from 0 (not at all) to 4 (very much), with a time interval of “within the last 2 weeks.” The scores for all 7 questions are summed, and the total score ranges from 0 to 28 points. A higher score indicates higher severity of insomnia.

To screen for hearing problems and sound tolerance problems (hyperacusis) that may interfere with participation in the intervention, Tinnitus and Hearing Survey (THS) will be used [43]. The measure will be used pre-treatment for screening and post-treatment at week 8, to assess any possible changes in score from baseline. The measure includes three subscales: tinnitus (4 items), hearing problems (4 items), and hyperacusis (1 item and 1 open question). Answers can range from 0 (not a problem) to 4 (very big problem). The total scores for tinnitus and hearing problems subscales can range from 0 to 16. Participants report their experiences in the last week to assess their current experience rather than their past history.

To assess the changes in the frequency of experiencing a state of mindful awareness, the Mindful Attention Awareness Scale (MAAS) will be used [44]. Assessment will be done pre-treatment, post-treatment (week 8), and at follow-ups (3 months and 12 months post-treatment); changes in scores from baseline will be assessed. The scale consists of 15 statements. Each statement is rated on a 6-point Likert scale (1 = almost always; 6 = almost never). Higher scores indicate a tendency to experience a more frequent state of mindful awareness, and a total score is either a mean or a sum of all answers.

To assess the changes in participants’ positive and negative cognitions related to tinnitus, the Tinnitus Cognitions Questionnaire (TCQ) will be used [45]. Assessment will be done pre-treatment, post-treatment (week 8), and at follow-ups (3 months and 12 months post-treatment), and changes in scores from baseline will be assessed. The questionnaire consists of 26 questions. Each statement is scored on a 5-point scale from 0 to 4 according to how frequently the cognition is thought (from 0 for “never” to 4 for “very often”). Negative items are scored from 0 to 4 and positive items from 4 to 0. The total score ranges from 0 to 104. A high score indicates a tendency to engage in negative cognitions in response to tinnitus rather than positive ones.

#### Other outcome measures

To assess the expressiveness of personality traits (extraversion, neuroticism, openness to experience, conscientiousness, and agreeableness), the Big Five Inventory (BFI-10) will be used in the pre-treatment assessment [46]. The questionnaire consists of 10 questions, two items per personality trait. Each statement is scored on a 5-point Likert scale (1 = strongly disagree and 5 = strongly agree). The higher the scores on the subscales, the more the personality trait is expressed.

Sociodemographic, tinnitus, and health-related questions will be assessed at pre-treatment. Questions to assess the eligibility for the study and to gather demographic and other important information will be used.

### Participant timeline {13}

Participant timeline is presented in Table 1.

**Table 1:** Schedule of enrollment, interventions, and assessments

Assessment/activity	Online screening/baseline	Telephone interview	Post-randomization			Follow-up	
	* − t*1	*t*0	*t*1–*t*8 (weekly)	*t*1–*t*8 (biweekly)	*t*9 (post)	*f*1	*f*2
Informed consent	X						
Demographic data	X						
iCBT			X				
iMBTSR			X				
THI-S				X			
THI	X				X	X	X
PHQ-9	X				X	X	X
GAD-7	X				X	X	X
ISI	X				X	X	X
THS	X				X		
MAAS	X				X	X	X
TCQ	X				X	X	X
BFI-10	X						
Eligibility criteria		X					
Treatment expectancy		X					
Treatment engagement			X				

### Sample size {14}

In order to attain 80% power with a 5% alpha level, a three-means non-inferiority test (ANOVA) was performed, resulting in a required sample size of 42 participants in total. To account for potential dropouts, the number of participants was increased by 25%, resulting in approximately 18 participants per study arm. This dropout rate is common for therapist-guided internet-based interventions. The primary end point used for the non-inferiority test was the THI score at 8 weeks, and the equivalence margin was determined to be 7 points, based on the mean minimally clinically important difference (MCID) found in a prior study [47].

### Recruitment {15}

The recruitment process will focus on the Lithuanian-speaking population and will utilize various methods to ensure sufficient participant enrollment. Announcements about the study will be made at tinnitus and hearing-related support groups, audiology organizations, and their newsletters and hearing clinics, as well as audiology departments and through social media. Interested individuals will be able to access information and register for the study through the study website.

### Assignment of interventions: allocation

#### Sequence generation {16a}

The principal investigator (PI) will utilize a random number generator (random.org) to allocate all participants randomly and equally to the study groups without stratification.

#### Concealment mechanism {16b}

The randomization sequence will not be concealed from the team member responsible for treatment allocation. Randomization sequence list will be generated once recruitment is finalized and added to a list of codes generated for each participant upon registration. Adding these two lists together will result in participants being allocated to study groups. The team member responsible for treatment allocation will not be able to identify any information about the participant from the code.

#### Implementation {16c}

Once the recruitment is complete, a principal investigator of the study will generate an allocation sequence using a random list generator www.random.org. The list will contain the number of items corresponding to the size of recruited participants. For example, if 60 participants will be recruited, then a list with 60 letters (20 letters a (a = treatment group no 1), 20 letters b (b = treatment group 2), and 20 letters c (c = waiting list control) arranged in a random order will be produced. This list will be added side-by-side to a list of alphabetically arranged codes (no personal information) generated to each participant upon registration.

### Assignment of interventions: blinding

#### Who will be blinded {17a}

Because of the psychological nature of the intervention, it is not possible to maintain blinding for either the participants or the study team members following the assignment to their respective intervention groups. All outcomes will be assessed via self-report measures which eliminate assessor bias. Data analysts will not be blinded because data output will be generated automatically from the study platform and will contain information specific to different study groups (completed modules, etc.) and will allow identification of assigned groups.

#### Procedure for unblinding if needed {17b}

The design is open label so unblinding will not occur.

### Data collection and management

#### Plans for assessment and collection of outcomes {18a}

Data will be collected at pre-randomization, termination, and 3- and 12-month follow-ups. All questionnaires will be self-report and administered online. To ensure that collected data is complete and accurate, the online questionnaires will be coded in a way that requires participants to fully answer the questions in order to submit the answers. For a full timeline of questionnaire dissemination, see Table 1.

#### Plans to promote participant retention and complete follow-up {18b}

All participants will be informed in the informed consent about follow-up assessments. If participants discontinue or deviate from intervention protocols, they will be contacted and study team members will first try to address any concerns participants might have that are altering their adherence to the intervention protocols. In case these concerns cannot be resolved, participants will then be asked to fill in follow-up self-assessment questionnaires online.

#### Data management {19}

Most of the data gathered online and saved to a database will be coded automatically. Only a small fraction of demographic data will need to be coded. Data will be stored on a secure server and, after the termination of the study, uploaded to MIDAS, a secure national scientific data repository, where it will be available upon request to the corresponding author after reports are published.

#### Confidentiality {27}

After giving informed consent, potential and enrolled participants will be asked to provide their email addresses and telephone numbers. The platform generates a unique code for each registered individual and stores it on a secure server available only to the research team with administrative rights after logging. Only non-personal data will be available from the data repository after the trial.

#### Plans for collection, laboratory evaluation, and storage of biological specimens for genetic or molecular analysis in this trial/future use {33}

Not applicable, no samples were collected.

## Statistical methods

### Statistical methods for primary and secondary outcomes {20a}

The differences between the groups at baseline will be evaluated using independent samples *t*-tests for continuous variables and chi-square tests for categorical variables or their non-parametrical equivalents. In order to assess the potential changes in the primary and secondary outcomes and compare them between the intervention groups and the control group, repeated-measures ANOVAs will be performed. The results from all baseline assessment measures will be utilized as predictors. The study will calculate both within-group and between-group effect sizes. The between-group effect sizes will be determined by calculating the mean difference from the pre-test to the post-test for short-term effects and from the pre-test to follow-up for long-term effects, using the standard deviations of each group at the pre-test. The within-group effect sizes will be calculated using the means and standard deviations at all time points for every group. We will use regression analysis to investigate the association between baseline variables and outcome measures, with the goal of identifying predictors of the outcome.

### Interim analyses {21b}

No interim analyses are planned.

### Methods for additional analyses (e.g., subgroup analyses) {20b}

Subgroup analyses are not planned.

### Methods in analysis to handle protocol non-adherence and any statistical methods to handle missing data {20c}

The results will be calculated based on the per-protocol approach and will also be compared with those obtained using the intention-to-treat approach. To perform the intention-to-treat analysis, a missing value analysis will be carried out. If necessary, missing data will be filled in using multiple imputation techniques.

### Plans to give access to the full protocol, participant-level data, and statistical code {31c}

De-identified research data will be stored and accessible to the principal investigator (PI) and available to those interested upon reasonable request.

### Oversight and monitoring

#### Composition of the coordinating center and trial steering committee {5d}

This trial does not include a coordinating center or trial steering committee. The sole responsibility for coordination falls on the core study team. Automated data collection and recorded database actions ensure data integrity. Any major changes to the study protocol will be reviewed and verified by the Research Ethics Committee. A physician with expertise in audiology and clinical experience will be available for consultation during the trial.

#### Composition of the data monitoring committee, its role, and reporting structure {21a}

A data monitoring committee is not required as all participants are self-referred and data is coded directly into a system that cannot be adjusted with all actions being logged. Weekly contact with research team members will be maintained to monitor for adverse events, which will be reported to the principal investigator. In case of adverse events, the participant will be interviewed by a clinically experienced team member and informed about relevant community services.

#### Adverse event reporting and harms {22}

Participants will be instructed that during the trial, they are to report any adverse effects to the trial administrator. A clinician from the research team will investigate any reported incidents and will direct participants to appropriate care if needed. All data on adverse events will be included in the publication of the main outcome results.

#### Frequency and plans for auditing trial conduct {23}

Sponsoring institution does not have a dedicated trial auditing department; therefore, no auditing is planned for this trial. Principal investigators will meet weekly during the study to review trial progress. Investigators are obliged to and will inform the research ethics committee in case of any major deviations from the study design approved by the ethics committee.

#### Plans for communicating important protocol amendments to relevant parties (e.g., trial participants, ethical committees) {25}

Before making any changes to the study protocol, approval from the Vilnius University Research Ethics Committee must be obtained. If approved, the changes must be reported in the trial register and included in the final report of research data.

#### Dissemination plans {31a}

The results of the trial will be published in a peer-reviewed journal and presented at scientific conferences. A popular science paper will be created for wider dissemination. To engage with the public, a public event will be organized to present the results. After the publication of the trial results, anonymized dataset will be available from PI upon reasonable request.

## Discussion

Internet-delivered psychological interventions for tinnitus provide several benefits including convenience, flexibility, reduced stigma, cost-effectiveness, and potential for scalability. Offering multiple effective options for Internet-delivered psychological treatments can improve access to care and lead to better outcomes for individuals with tinnitus. More specifically, by providing a wider range of choices to better fit individual preferences, different approaches to target specific needs, easier access to care especially for those in remote areas, and ultimately, better outcomes. These factors can improve the overall quality of life for people with tinnitus. Moreover, empowering individuals to choose between multiple options can increase engagement in treatment, improve the sense of control, and enhance adherence to treatment, leading to better outcomes. The findings of this study could add to the empirical evidence of the efficacy of CBT and mindfulness-based interventions.

Overall, Internet-delivered psychological interventions might provide an accessible, flexible, and convenient way to receive evidence-based treatment for tinnitus.

## Trial status

Protocol version 1, 2022–11-13. Recruitment did not yet commence. Recruitment is planned to begin mid-February 2023 and extend into the first half of March 2023.

## Data Availability

After the publication of trial results, a de-identified dataset will be available from the PI upon a reasonable request.

## Abbreviations

CBT: Cognitive behavioral therapy
CONSORT: Consolidated Standards of Reporting Trials
ICBT: Internet-delivered cognitive behavioral therapy
iMBTSR: Internet-delivered mindfulness-based tinnitus stress reduction
ITT: Intention-to-treat
PI: Principal investigator
PPA: Per-protocol analysis
RCT: Randomized controlled trial
